# Potential use of telephone surveys for non-communicable disease surveillance in developing countries: evidence from a national household survey in Lebanon

**DOI:** 10.1186/s12874-016-0160-0

**Published:** 2016-05-31

**Authors:** Abla M. Sibai, Lilian A. Ghandour, Rawan Chaaban, Ali H. Mokdad

**Affiliations:** Department of Epidemiology and Population Health, Faculty of Health Sciences, American University of Beirut, Beirut, Lebanon; Institutes for Health Metrics and Evaluation, University of Washington, Seattle, WA USA

**Keywords:** Cell phones, Landline phones, Risk factors, Noncoverage bias, NCD, Lebanon

## Abstract

**Background:**

Given the worldwide proliferation of cellphones, this paper examines their potential use for the surveillance of non-communicable disease (NCD) risk factors in a Middle Eastern country.

**Methods:**

Data were derived from a national household survey of 2,656 adults (aged 18 years or older) in Lebanon in 2009. Responses to questions on phone ownership yielded two subsamples, the ‘cell phone sample’ (*n* = 1,404) and the ‘any phone sample’ (*n* = 2,158). Prevalence estimates of various socio-demographics and 11 key NCD risk factors and comorbidities were compared between each subsample and the overall household sample.

**Results:**

Adjusting for baseline age and sex distribution, no differences were observed for all NCD indicators when comparing either of subsamples to the overall household sample, except for binge drinking [(OR = 1.55, 95 % CI: 1.33–1.81) and (OR = 1.48, 95 % CI: 1.18–1.85) for ‘cell phone subsample’ and ‘any phone subsample’, respectively] and self-rated health (OR = 1.23, 95 % CI: 1.10–1.36) and (OR = 1.16, 95 % CI: 1.02–1.32), respectively). Differences in the odds of hyperlipidemia (OR = 1.27, 95 % CI: 1.06–1.51) was also found in the subsample of ‘any phone’ carriers.

**Conclusions:**

Multi-mode telephone surveillance techniques provide viable alternative to face-to-face surveys in developing countries. Cell phones may also be useful for personalized public health and medical care interventions in young populations.

## Background

Low and middle income countries, worldwide and in the Arab region, are undergoing a rapid rise in the burden of non-communicable diseases (NCDs) with major adverse economic and health impacts [1, 2]. Yet, capacity for prevention and control, including national monitoring and disease-surveillance frameworks, remains inadequate particularly in resource-scarce countries. Consequently, periodic population-based surveys have been advocated as an entry point to provide cross-sectional estimates of prevalence rates of NCDs and their risk factors. These surveys have traditionally relied on face-to-face household interviews, which are both costly and time-consuming [3]. Alternatively, random digit dialing (RDD) sampling and landline telephone surveys have become increasingly used in health surveys, notably in the Americas, for monitoring NCD risk factors among the general population (e.g. the CDC Behavioral Risk Factor Surveillance System in the US and the Telephone-based Surveillance of Risk and Protective Factors for Chronic Diseases in Brazil [4, 5]). Compared to household sampling, surveys using RDD are typically faster and allow greater access to respondents, regardless of their residence area or daily schedules [6]. Also, interviews using landline phones are less intrusive, ensure greater anonymity and minimize social desirability bias and interviewer effects [7, 8].

Today, however, with the worldwide exponential increase in cell phone usage and concomitant trend of trading landline for cell phone services, the external validity of the landline telephone sampling frames has become questionable [8, 9]. Recent U.S. data show that nearly 39 % of American homes do not own a landline and 1 out of 6 receive almost all calls on cell phones despite owning a landline, increasing the percentage of those ‘unreachable via a landline’ to approximately 55 % [10]. In Europe, the share of mobile-only households ranges from 3 % in Sweden, to 23 % in Ireland, and 64 % in the Czech Republic [11]. This shift presents a number of methodological challenges to landline RDD, notably noncoverage bias [8, 9, 12]. This form of selection bias arises from failure to include some elements of the target population, threatening the representativeness of the sample. Still, a high noncoverage rate does not guarantee high bias, and the reverse is true. Noncoverage bias may be trivial if those who are covered by the sampling frame are similar to those who are not in terms of the variables of interest [13].

Despite the rising prevalence of cell phone ownership worldwide, including developing countries, and the increase in wireless households among all social and economic segments of the world population, published studies on cell phones interviews remain only a handful from outside the Americas [11, 14–16]. Today, coverage rates for the cell phone market have already exceeded 100 % (multiple cell phones per subscriber) in several parts of the world, including the Middle East and North African (MENA) region (e.g. 153 % in Qatar and 172 % in the United Arab Emirates [17]). In Lebanon, a small middle-income country in the MENA region (around 4 million population and a Gross Domestic Product of 9,284 USD per capita), the number of cell phone subscriptions has more than doubled in recent years (34 % in 2008, 66 % in 2010 and 81 % in 2013). This rise in cellphone usage in Lebanon offers an appealing alternative to face-to-face interviews, especially that in Lebanon (and other parts of the region) access to certain geographical areas may be physically challenging due to conflict or presence of gated communities, and people may have become more reluctant to let in strangers into their homes given security concerns [unpublished observations, Ghandour et al.].^1^

Using data from the nationwide *Nutrition and Non-Communicable Disease Risk Factor Survey* (NNCD-RFS) in Lebanon, the present paper aims to examine in a non-western context the magnitude and elements of noncoverage bias when employing a ‘cell phone’ framework or a dual ‘cell phone and landline’ framework compared to a household sampling frame. More specifically, our objective is to compare the population-based estimates of key NCD indicators between each cell phone and any phone subsamples with household survey results. The ultimate goal is to estimate the potential resulting non-coverage bias, and examine the extent to which adjustment by age and sex reduce differentials and, hence, noncoverage bias.

## Methods

### Study design and participants

Data were drawn from the NNCD-RFS nationwide cross-sectional population-based study conducted in Lebanon in 2009 by the Population Health and Nutrition Group at the American University of Beirut (AUB). The study followed the World Health Organization (WHO) STEPwise survey methodology and was approved by the AUB Institutional Research Board. The study sample was based on the sampling frame provided by the National Survey of Household Living Conditions, which was conducted by the Ministry of Social Affairs/Central Administration of Statistics in collaboration with United Nations Development Programme (UNDP). Households were randomly selected using multi-stage cluster proportionate probability sampling. Face-to-face interviews were conducted with one randomly selected adult (using the Kish-method). The final number of respondents (2,836) was comparable by age, sex and district distribution to the overall target population of residents in Lebanon [18]. Hence our study is based on a nationally representative household (HH) sample of adults generated from a household face-to-face survey. Respondents were asked whether the household they lived in has a landline telephone and whether they personally owned a cellphone. Subjects with missing information on phone ownership (*n* = 180) were excluded from the study, yielding a final sample of 2,656 for the ‘original HH sample’ available for this analysis. The majority of the HH sample (*n* = 2,126; 80.0 %) had either a cellphone or a landline telephone (i.e. ‘any phone sample’); and about half (*n* = 1,381; 52.0 %) had a cell phone, irrespective of landline ownership (i.e. ‘cell phone sample’). More specifically, 780 respondents had a cell phone only, 744 had a landline only, and a total of 602 respondents had both telephone types.

### Measures

Our survey included questions on socio-demographic variables and household characteristics. These included sex, age, marital status (single/married/divorced or widowed), governorate of residence (Beirut/Mount Lebanon/North/South/Bekaa/Nabatieh), education level (primary and below/complementary/secondary or technical/university and above), occupational status (do not work/governmental employee/non-governmental employee/self-employed), crowding index, home ownership (yes/no) and insurance coverage (yes/no). We created a crowding index reflecting the number of household members divided by the number of rooms excluding kitchens, bathrooms and non-closed balconies.

Moreover, and following the STEPwise interview schedule protocol, the survey included questions on NCD risk factors. For this analysis, 11 key self-reported NCD risk factors and comorbidities were considered: current tobacco consumption (cigarette and waterpipe), past-month binge drinking, hypertension, diabetes, hyperlipidemia, cardiovascular disease, asthma, weight and height (yielding BMI measure), disability based on activities of daily living index, and self-rated health. Tobacco consumption was assessed by asking respondents, “Do you currently smoke any tobacco products, such as cigarettes, cigars, pipes or Shisha/water pipe?” We focus here on current cigarette smoking and current water pipe smoking. Binge drinking was defined as consuming five or more drinks for males and four or more drinks for females in one sitting at least once in the preceding month. Reporting of health conditions was based on a question that inquired whether respondents had ever been diagnosed with the “disease” by a health professional. Cardiovascular disease included heart disease, peripheral arterial disease, myocardial infarction or stroke. Body mass index (BMI) was calculated as the ratio of weight (kilograms) to square of height (meters), and obesity was defined as a BMI of greater than 30 kg/m^2^. Finally, self-rated health was assessed through a single question asking respondents to rate their overall health on a 5-point scale (excellent, very good, good, poor or very poor); responses were dichotomized as very good and/or excellent versus good and/or worse.

### Analysis

The percent distribution of the different measures was compared between each of the two subsamples (i.e. ‘any phone’ and ‘cell phone’) with the ‘original HH sample’ using the one-sample test for proportion. Logistic regression with ‘deviation coding’ was run, and both unadjusted and adjusted odds ratios (OR) and 95 % confidence intervals (CI), controlling for age and sex, are presented (uOR and aOR, respectively). Both the one-sample test for proportion and the ‘deviation coding’ assume that the subsample estimate is the ‘sample statistic’ and that of the household survey is the ‘population parameter’. Using the direct adjustment method, the prevalence rates of the various NCD and health indicators derived from the two subsamples (i.e. ‘cell phone sample’ and ‘any phone sample’) were finally re-estimated based on the age and sex distribution of the ‘original HH sample’. All analyses were weighted, and were run using SPSS (version 16.0) and Stata MP (release 12).

## Results

### Socio-demographic differences

A comparison of the socio-demographic characteristics of respondents belonging to each of the subsamples (‘cell phone carriers’ and ‘any phone carriers’) and the HH sample is presented in Table 1. Those with cellular phones were more likely to be male, young, single, from Beirut (the capital city), of a higher educational level, part of the labor force, living in a less crowded household, and insured. The largest deviation in the estimates was observed for insurance coverage (absolute difference of 20.9 %), followed by occupational status (16.7 %), education (13.0 %), thereafter age, gender and marital status (range of absolute difference: 11.3–12.8 %). With regards to ‘any phone sample’ (compared to the HH sample), the differences by gender, education, occupational status, house ownership, crowding index,, although statistically significant, were very subtle (range of absolute difference: 3.0–4.2 %). Percent difference (19.3 %) for insurance coverage was however more obvious. Generally, however, larger absolute differences in prevalence estimates for sociodemographics were observed for the ‘cell phone sample’ but not for the ‘any phone sample’ when compared to the ‘original HH sample’; hence, a larger noncoverage bias for the ‘cell phone sample’ subgroup.

**Table 1:** Comparison of the sociodemographic characteristics of the ‘cell phone carrier’ and the ‘any phone carrier’ subsamples to the ‘original HH sample’

	Original HH sample (A)		Cell phone sample^a^ (B)		Any phone sample^b^ (C)		*p*-value (B vs. A)	*p*-value (C vs. A)
	N_A_	%	n_B_	%	n_C_	%		
Total	2656	100	1381	52.0	2126	80.0		
Gender								
Male	1238	46.6	799	57.9	1054	49.6	<0.001	<0.001
Female	1418	53.4	582	42.1	1072	50.4		
Age Group								
18–34 years old	1237	46.6	820	59.4	1003	47.2	<0.001	0.602
35–54 years old	961	36.2	421	30.5	747	35.2	<0.001	0.310
≥ 55 years old	456	17.2	140	10.1	373	17.6	<0.001	0.666
Marital Status								
Single	1069	40.3	723	52.4	881	41.5	<0.001	0.289
Married	1424	53.6	605	43.8	1113	52.4	<0.001	0.249
Divorced/widowed	163	6.1	53	3.8	131	6.2	<0.001	0.891
Governorate								
Beirut	273	10.3	197	14.3	239	11.2	<0.001	0.154
Mount Lebanon	979	36.8	540	39.1	772	36.3	0.078	0.653
North	601	22.6	282	20.4	454	21.4	0.054	0.178
South	289	10.9	152	11.0	244	11.5	0.897	0.384
Bekaa	349	13.1	146	10.6	286	13.5	0.004	0.629
Nabatieh	165	6.2	64	4.6	130	6.1	0.014	0.928
Education								
Primary and below	545	20.5	131	9.5	354	16.7	<0.001	<0.001
Complementary	663	24.9	302	21.9	508	23.9	0.008	0.292
Secondary/Technical	681	25.7	369	26.7	563	26.5	0.388	0.413
University & above	767	28.9	579	41.9	701	33.0	<0.001	<0.001
Occupational Status								
Do not work	1426	53.7	511	37.0	1052	49.5	<0.001	<0.001
Governmental employee	165	6.2	111	8.0	151	7.1	0.080	0.087
Non- Governmental employee	515	19.4	373	27.0	438	20.6	<0.001	0.162
Self-employed	550	20.7	386	28.0	484	22.8	<0.001	0.019
Crowding Index								
< 1 person per room	692	26.1	436	31.6	634	29.9	<0.001	<0.001
≥ 1 person per room	1955	73.9	942	68.4	1484	70.1		
House Ownership								
Owned	1885	71.0	984	72.1	1550	73.7	0.647	<0.001
Rented	744	28.0	381	27.9	552	26.3		
Insurance coverage								
Covered	1139	42.9	880	63.7	1321	62.2	<0.001	<0.001
Uncovered	1517	57.1	501	36.3	804	37.8		

^a^‘Cell phone sample’ included those who reported owning a cell phone, irrespective of landline ownership

^b^‘Any phone sample’ included those who reported having a cell phone or a landline

### Health indicators

The prevalence estimates for the NCD behavioral and health indicators were for the most part (9 out of 11 indicators, except for cigarette smoking and asthma) statistically different between the ‘cell phone sample’ and the ‘original HH sample’ at the unadjusted level (Table 2, uOR). Quite importantly however, the magnitude of the differences was attenuated and findings were no longer statistically significant when adjusted for age and sex, except in the case of two indicators, binge drinking and self-rated health (Table 2, aOR). This was further illustrated by direct adjustment of the estimates by age and sex, whereby percent differences were reduced to less than 2.0 % for all variables except for binge drinking and self-rated health (3.2 % and 2.9 %, respectively). Differences for two additional health indicators (high blood pressure and cardiovascular diseases) were also reduced by about 35 % but remained borderline statistically significant (Table 2).

**Table 2:** Prevalence estimates, odds ratios (OR) and 95 % confidence intervals (CI) comparing the ‘cell phone sample’ to the ‘original HH sample’

NCD and public health indicators	Prevalence (%) in original HH sample (A)	Prevalence (%) in cell phone sample (B)	uOR^c^	95 % CI	aOR^c^	95 % CI	Age-sex adjusted prevalence (%) in cell phone sample
Behaviors							
Current cigarette smoking	34.7	35.5	1.04	0.95–1.13	1.06	0.96–1.17	36.2
Current water pipe smoking	25.0	29.8	1.31*	1.18–1.45	1.09	0.98–1.22	26.2
Binge drinking in past 30 days^a^	11.7	17.4	1.87*	1.62–2.17	1.55*	1.33–1.81	14.9
Health Conditions							
High Blood Pressure	14.0	8.7	0.62*	0.55–0.70	0.85*	0.74–0.98	11.9
Diabetes	6.4	5.2	0.82*	0.69–0.97	1.13	0.93–1.37	6.9
Hyperlipidemia	13.3	11.2	0.83*	0.73–0.93	1.10	0.97–1.26	14.2
Cardiovascular disease	7.0	4.1	0.62*	0.52–0.73	0.82*	0.67–0.99	5.2
Asthma	6.1	6.4	1.05	0.88–1.25	1.05	0.87–1.28	6.5
Obesity (BMI ≥ 30 kg/m^2^)	24.4	20.7	0.80*	0.72–0.88	0.93	0.84–1.04	23.2
Disability	1.8	1.3	0.72*	0.53–0.99	0.90	0.63–1.29	1.5
Self-Rated Health^b^	32.0	39.1	1.51*	1.33–1.62	1.23*	1.10–1.36	34.9

^a^Binge drinking is defined as consuming five or more drinks (male) or four or more drinks (female) at least once in past 30 days

^b^very good and better versus good and worse

^c^uOR: unadjusted odds ratios comparing ‘cell phone sample’ to total household population sample; aOR: odds ratios adjusted for age and sex

*Asterisk indicate statistically significant results (*p* value < critical alpha of 0.05)

Comparing the ‘any phone sample’ to the overall population at the unadjusted level, differences were noted for a lesser number of indicators (4 out of 11; Table 3, uOR), which did not change appreciably after controlling for age and sex; specifically, differences in estimates of binge drinking, hyperlipidemia, and self-rated health remained statistically different even after adjustment (Table 3, uOR). In summary, adjusting for age/sex reduced the differences in the estimates in the case of the cellphone to 2 indicators (suggesting that most differences can be accounted for by age and sex adjustment), whereas the number of differences in the any phone sample was not affected.

**Table 3:** Prevalence estimates, odds ratios (OR) and 95 % confidence intervals (CI) comparing the ‘any phone sample’ to the ‘original HH sample’

NCD and public health indicators	Prevalence (%) in original HH sample (A)	Prevalence (%) in any phone sample (C)	uOR^a^	95 % CI	aOR^a^	95 % CI	Age-sex adjusted prevalence (%) in any phone sample
Behaviors							
Current cigarette smoking	34.7	34.7	1.00	0.89–1.11	0.95	0.85–1.07	34.8
Current water pipe smoking	25.0	25.5	1.06	0.93–1.21	1.05	0.92–1.19	24.3
Binge drinking in past 30 days^b^	11.7	13.3	1.63*	1.31–2.02	1.48*	1.18–1.85	12.5
Health Conditions							
High Blood Pressure	14.0	13.4	0.89	0.76–1.03	0.85	0.72–1.01	15.3
Diabetes	6.4	6.8	1.26*	1.01–1.58	1.25	0.99–1.56	7.7
Hyperlipidemia	13.3	14.2	1.23*	1.04–1.46	1.27*	1.06–1.51	15.7
Cardiovascular disease	7.0	7.2	1.10	0.88–1.37	1.07	0.83–1.38	8.3
Asthma	6.1	6.0	0.94	0.75–1.18	0.94	0.75–1.19	6.0
Obesity (BMI ≥ 30 kg/m^2^)	24.4	24.3	0.95	0.83–1.07	0.95	0.83–1.08	25.6
Disability	1.8	1.7	0.84	0.60–1.18	0.78	0.54–1.12	1.9
Self-Rated Health^c^	32.0	33.1	1.23*	1.09–1.39	1.16*	1.02–1.32	31.6

^a^uOR: unadjusted odds ratios comparing ‘any phone sample’ to total household population sample; aOR: odds ratios adjusted for age and sex

^b^Binge drinking is defined as consuming five or more drinks (male) or four or more drinks (female) at least once in past 30 days

^c^very good and better versus good and worse

*Asterisk indicate statistically significant results (*p* value < critical alpha of 0.05)

## Discussion

Our nationally representative study is the first to examine the noncoverage bias associated with the use of telephone surveys for NCD surveillance in Lebanon and the region. The findings presented are of great relevance to the Arab World and developing countries. They provide evidence that phone-based surveys are likely to offer a viable alternative to the expensive and time-consuming household surveys, and could also be used to provide real-time monitoring of health events at the national level.

Using the same dataset, we had previously shown that using telephone surveys may provide a valid and cheaper alternative to household interviews [19]. We were able to demonstrate a high level of agreement in responses and information gathered using cell phones and face-to-face interviews, while saving approximately $14 per participant (close to 35 % less cost) in cell phone interviews [19]. Findings from this present study further indicate that while cell-phone sampling frames may yield samples that are socio-demographically different from household-based samples, ultimately the magnitude of the differences in the prevalence rates of various NCD indicators becomes negligible once adjusted by age and sex distribution of the overall target population. This is similar to previous findings reported in the West [12, 20]. Specifically, adjusting for two basic demographic characteristics (age and sex) reduced the percentage point difference between the subgroup and the target population for the majority of NCD indicators, hence mitigating noncoverage bias. Altogether, cellphone surveys seem to have great potential for enabling decision makers in developing countries to collect data in a timely manner.

Still, biased estimates for certain health indicators, specifically binge drinking and self-rated health, may continue to exist even after age-sex adjustment. The inflated estimates of binge drinking in both subsamples (‘cell phone’ and ‘any phone’) are likely to be explained by the ‘wireless lifestyle’ [21], serving as the driving force for an active social life associated with binge drinking. Post hoc analyses of our data showed that cell phone-only adults (*n* = 780) reported the highest rates of binge drinking (19.7 %), followed by the ‘any phone’ subgroup (13.3 %), then the landline-only respondents (5.8 %). Indeed, the most consistent finding in the literature examining cell phone-only generation is that they tend to be young and to have distinctively higher rates of smoking and binge drinking, re-enforcing the fact that significant noncoverage bias would be introduced if the subpopulation of cell phones only were not included in a RDD sampling frame [9, 22, 23]. Our results are also relevant to the more recent worldwide developments encouraging the use of mobile health (mHealth) applications [24], when trying to reach high risk populations for health promotion purposes (e.g., binge drinkers in our case), or for treatment compliance and appointment reminders.

Furthermore, our finding that self-rated health remained different in both comparisons (even after adjustment by age and sex) may be due to the fact that perception of one’s health is subjective in nature and perhaps is very sensitive to a variety of social, psychological and physical health aspects as well as broader resources such as financial and economic well-being [25]. Until future research further examines and better understands the underlying reasons for these observed differences, our finding should discourage researchers from attempting to investigate subjective measures of health in future phone surveys.

### Limitations

Our findings should be interpreted in light of some study limitations. First, the estimates provided in this paper reflect ‘theoretically’ what would be the noncoverage bias if cell phones or dual phone lines were used instead of face-to-face household interviews, and do not represent actual findings from telephone-based sampling. Our study is also based on self-reported data of phone ownership and may be subject to information bias. In the adjusted analyses, we presented findings controlling for only respondents’ age and sex distributions, because further analyses [available upon request] additionally controlling for marital status, education and governorate of residence yielded essentially similar results. Thus, we opted to present findings estimates from the more parsimonious model in keeping with the larger goal of this research, which is to examine the practical viability of conducting phone surveys in challenging contexts without jeopardizing the validity of the survey estimates. From a practical point of view, national data on age and sex may be easier to obtain in contrast to data on additional confounders.

Telephone surveys also have their own challenges such as nonresponse bias, particularly that response rates have been on a precipitous decline [26]. However, the decline in response rate is becoming a major concern for all modes of data collection, including household interviews [27]. Yet, cellphone nonresponse issues may be more challenging in some settings than others. Previously, we showed that these issues do not constitute a serious threat to RDD surveying in Lebanon: of the 771 cell phone numbers contacted for a re-interview, only 4 % were not reachable because the cell phone number was temporarily or permanently out of service, 5 % were found to belong to other than the selected participant, and 8 % refused to participate [19]. Moreover, an important and highly relevant aspect to cellphone surveys is the fact that incoming calls in Lebanon are free of any charge, unlike in other contexts where respondents may be charged for incoming calls [28]; hence, in Lebanon, respondents would bear no financial repercussions if they were to participate in the telephone survey, a factor that is likely to reduce non-response rate.

## Conclusions

In Lebanon as in many other countries, cellphone coverage is increasing and cellular phones are reaching a wide range of socio-demographic subgroups in the population. Hence, health surveys based on cellular phones in developing countries have the potential of reaching a representative sample of the population. This is contrary to landlines, which are becoming more expensive and a mode of telecommunication for only a select group of general population [i.e. would not represent on their own an adequate sampling frame especially for younger adults].

Our study suggests that cellphone surveys could be integrated into current existing systems for NCD surveillance and health monitoring. It also suggests that we may reach different segments of the population when using different data collection modes in population-based surveys, and that cell phone surveys, in particular, may be useful in reaching at risk young populations, supporting the use of mobiles for personalized public health and medical care interventions. The fact remains that certain individuals may still prefer one method over the other and it may be time to accept these differences and apply a multi- mode system for national surveillance. Indeed, telephone surveys may be conducted on a yearly basis while household surveys less frequently. Data from both sources when conducted in the same year could be used to adjust for bias in the telephone surveys in the years between.

Multi-mode telephone surveillance techniques provide viable alternative to face-to-face surveys in developing countries. As the demand for regular health data increases in the region and elsewhere, more research is needed to examine the feasibility and validity of dual-mode or multi-mode surveillance techniques in health research or the proper surveillance and monitoring of major health indicators.

### Availability of data and materials

The study is based on secondary data analysis NNCD-RFS study. All data supporting our findings are contained within the manuscript. The original data set is owned by the first author and is made available to researchers and students within the Faculty of Health Sciences at the American University of Beirut. Researchers interested in using these data can contact the first author with a proposal and specific research questions.

### Ethics

The original study was granted ethical approval by the Institutional Review Board of the American University of Beirut.

### Consent to participate

Participants read and signed a written informed consent form.

### Consent to publish

Not applicable.

## Abbreviations

aOR: adjusted odds ratios
AUB: American University of Beirut
BMI: Body Mass Index
CDC: Centers for Disease Control and Prevention
CI: confidence interval
HH: household
MENA: Middle East and North Africa
mHealth: Mobile Health
NCD: Non-Communicable Disease
NNCD-RFS: Nutrition and Non-Communicable Disease Risk Factor Survey
ORs: odds ratios
RDD: random digit dialing
SPSS: Statistical Package for the Social Sciences
U.S.: United States
uOR: unadjusted odds ratios
USD: United States Dollar
WHO: World Health Organization

## Footnotes

1 Ghandour L, Shehab S, Zeinoun P, Halabi F, Maalouf F: Against all odds: Contextual Challenges and Implications to Undertaking a Household Adolescent Mental Health Survey in a Developing country. (Submitted, BMC Psychiatry)

## References

[1] Rahim HFA, Sibai AM, Khader Y, Hwalla N, Fadhil I, Alsiyabi H, Mataria A, Mendis S, Mokdad AH, Husseini A (2014). Non-communicable diseases in the Arab world. Lancet.

[2] Mokdad A, Jaber S, Abdel Aziz M, AlBuhairan F, AlGhaithi A, AlHamad NM, Al-Hooti SN, Al-Jasari A, AlMazroa MA, AlQasmi AM, Alsowaidi S, Asad M, Atkinson C, Badawi A, Bakfalouni T, Barkia A, Biryukov S, El Bcheraoui C, Daoud F, Forouzanfar MH, Gonzalez-Medina D, Hamadeh RR, Hsairi M, Hussein SS, Karam N, Khalifa SEAH, Khoja TAM, Lami F, Leach-Kemon K, Memish ZA, Mokdad AA, Naghavi M, Nasher J, Qasem MBH, Salman TA, Shuaib M, Al Thani AAM, Al Thani MH, Zamakhshary M, Lopez AD, Murray CJ L (2014). The state of health in the Arab world, 1990–2010: an analysis of the burden of diseases, injuries, and risk factors. Lancet.

[3] Aday LA, Cornelius LJ (2011). Designing and conducting health surveys: a comprehensive guide.

[4] Mokdad A (2009). The Behavioral Risk Factors Surveillance System: past, present, and future. Annu Rev Public Health.

[5] Malta DC, da Silva SA, de Oliveira PP, Iser BP, Bernal RT, Sardinha LM, de Moura L (2012). Monitoring of risk and protective factors for chronic non communicable diseases by telephone survey in Brazilian State Capitals, 2008. Rev Bras Epidemiol.

[6] Sturges JE, Hanrahan KJ (2004). Comparing telephone and face-to-face qualitative interviewing: a research note. Qual Res.

[7] Aziz M, Kenford S (2004). Comparability of telephone and face-to-face interviews in assessing patients with posttraumatic stress disorder. J Psychiatr Pract.

[8] Kempf AM, Remington PL (2007). New challenges for telephone survey research in the twenty-first century. Annu Rev Public Health.

[9] Link MW, Battaglia MP, Frankel MR, Osborn L, Mokdad AH (2007). Reaching the US cell phone generation: comparison of cell phone survey results with an ongoing landline telephone survey. Public Opin Q.

[10] Blumberg SJ, Luke JV. Wireless substitution: early release of estimates from the National Health Interview Survey, January–June 2013. National Center for Health Statistics; 2013. http://www.cdc.gov/nchs/nhis.htm. Accessed 02 Feb 2015

[11] Kühne M, Häder M, Häder S, Häder M, Kühne M (2012). Telephone surveys via landline and mobile phones: Mode effects and response quality. Telephone surveys in Europe.

[12] Lee S, Brick JM, Brown ER, Grant D (2010). Growing cell-phone population and noncoverage bias in traditional random digit dial telephone health survey. Health Serv Res.

[13] Lee S, Elkasabi M, Streja L (2012). Increasing cell phone usage among Hispanics: Implications for telephone surveys. Am J Public Health.

[14] Bradley J, Ramesh BM, Rajaram S, Lobo A, Gurav K, Isac S, Chandra ShekharGowda G, Pushpalatha R, Moses S, Sunil KD, Alary M (2012). The feasibility of using mobile phone technology for sexual behaviour research in a population vulnerable to HIV: a prospective survey with female sex workers in South India. AIDS Care.

[15] Song Z, Foo MD, Uy MA (2008). Mood spillover and crossover among dual-earner couples: a cell phone event sampling study. J Appl Psychol.

[16] McBride O, Morgan K, McGee H (2012). Recruitment using mobile telephones in an Irish general population sexual health survey: challenges and practical solutions. BMC Med Res Methodol.

[17] World Bank Group. Mobile cellular subscriptions (per 100 people), http://data.worldbank.org/indicator/IT.CEL.SETS.P2 (2014). Accessed 03 Jan 2015.

[18] Central Administration of Statistics. National Survey of Household Living Conditions: Report on Household Living Conditions, 2007. 2008. http://www.cas.gov.lb. Accessed 03 Jan 2015.

[19] Mahfoud Z, Ghandour L, Ghandour B, Mokdad A, Mehio Sibai A (2015). Comparison of cell phone and face-to-face interviews in population-based surveys. Field Methods.

[20] Hu SS, Balluz L, Battaglia MP, Frankel MR (2011). Improving public health surveillance using a dual-frame survey of landline and cell phone numbers. Am J Epidemiol.

[21] Blumberg SJ, Luke JV (2009). Reevaluating the need for concern regarding noncoverage bias in landline surveys. Am J Public Health.

[22] Blumberg S, Luke J (2007). Coverage bias in traditional telephone surveys of low-income and young adults. Public Opin Q.

[23] Delnevo CD, Gundersen DA, Hagman BT (2008). Declining estimated prevalence of alcohol drinking and smoking among young adults nationally: artifacts of sample undercoverage?. Am J Epidemiol.

[24] World Health Organization. mHealth: new horizons for health through mobile technologies. 2011. http://www.who.int/goe/publications/goe_mhealth_web.pdf. Accessed 01 Apr 2013.

[25] Chemaitelly H, Kanaan C, Beydoun H, Chaaya M, Kanaan M, Sibai AM (2013). The role of gender in the association of social resources and economic circumstances with self-rated health among older adults in underserved communities in Beirut. Qual Life Res.

[26] Curtin R, Presser S, Singer E (2005). Changes in telephone survey nonresponse over the past quarter century. Public Opin Q.

[27] Galea S, Tracy M (2007). Participation rates in epidemiologic studies. Ann Epidemiol.

[28] Brick JM, Brick PD, Dipko S, Presser S, Tucker C, Yuan Y (2007). Cell phone survey feasibility in the U.S.: sampling and calling cell numbers versus landline numbers. Public Opin Q.

